# Association of SNP Rs6903956 on Chromosome 6p24.1 with Angiographical Characteristics of Coronary Atherosclerosis in a Chinese Population

**DOI:** 10.1371/journal.pone.0043732

**Published:** 2012-08-29

**Authors:** Chang-Yan Guo, Yan Gu, Li Li, En-Zhi Jia, Chun-Jian Li, Lian-Sheng Wang, Zhi-Jian Yang, Ke-Jiang Cao, Wen-Zhu Ma

**Affiliations:** Department of Cardiovascular Medicine, First Affiliated Hospital of Nanjing Medical University, Nanjing, Jiangsu Province, China; Leibniz-Institute for Arteriosclerosis Research at the University Muenster, Germany

## Abstract

**Objective:**

To explore the association between rs6903956 and severity of coronary artery disease (CAD) in a Chinese population.

**Methods:**

A cohort of 1075 consecutive patients who underwent coronary arteriography for suspected or known coronary atherosclerosis was enrolled in our study. Coronary atherosclerosis severity was defined by Gensini's Score System and counts of diseased vessels.

**Results:**

Gensini score frequencies and counts of diseased vessels differed among GG, AG, AA genotype groups at the rs6903956 locus (*p* = 0.025 for Gensini score frequencies vs. *p* = 0.024 for counts of diseased vessels, respectively). A univariate logistic regression analysis revealed that the genotype distribution of this SNP was associated significantly with angiographical characteristics of coronary atherosclerosis risk (*p* = 0.030, odds ratio (*OR*) = 1.444, 95% confidence interval (*CI*) = 1.036∼2.013 for AG vs. GG; *p* = 0.021, *OR* = 5.896, 95% *CI* = 1.299∼26.750 for AA vs. GG and *p* = 0.007, *OR* = 1.564, 95% *CI* = 1.132∼2.162 for combined (AG+AA) vs. GG). A multivariate logistic regression analysis indicated that the genotype distribution of the rs6903956 polymorphism be associated significantly with the angiographical characteristics of coronary atherosclerosis risk (*p* = 0.004, *OR* = 1.578, 95% *CI* = 1.155∼2.154 for GG vs. AG vs. AA; *p* = 0.013, *OR* = 1.541, 95% *CI* = 1.097∼2.163 for GG vs. GA+ AA). A stratification analysis revealed that male subjects and smoking subjects had a higher frequency of the rs6903956 heterozygous mutant among higher Gensini score subjects than among lower Gensini score subjects (*p* = 0.023, *OR* = 1.579, 95% *CI* = 1.064∼2.344 for male subgroup; *p* = 0.005, *OR* = 2.075, 95% *CI* = 1.249∼3.448 for smoking subgroup).

**Conclusions:**

Allele A is a risk factor for CAD and the G-to-A allele substitution may underlie the association between rs6903956 and CAD.

## Introduction

Coronary artery disease (CAD) is predicted to be the most common cause of death globally, including in China, by 2020, when the 10 leading causes of disability-adjusted life-years (in descending order) are projected to be ischaemic heart disease [1], In addition to lifestyle and environmental factors, which are major aetiologic determinants, multiple combinations of gene–gene and gene–environment interactions play a key role in the development of CAD [2]–[3]. Although the heritability of CAD is estimated to be 40 to 60%, the mechanism of genetic is still little known [4]. A number of genomic regions, variants in candidate genes, and risk factors were implicated in increasing the susceptibility of CAD [5]. However, most of the variants and genes have not been established consistently. The most robust genetic risk variant for CAD was identified on chromosome 9p21.3 by genome-wide association studies (GWAS) [6]. Recently, the first GWAS for CAD in a Chinese Han population had identified rs6903956, which is in *C6orf105* on chromosome 6p24.1, to be significantly associated with susceptibility to CAD [7].

However, the true causative of rs6903956 to CAD is unknown. Despite its limitations, coronary arteriography remains to be the golden standard for documenting the extent and severity of CAD. Our research seek to test the hypothesis that 6p24.1 locus promote atherosclerosis by examining the relationship between rs6903956 genotype and angiographic severity in Chinese population who underwent coronary arteriography.

## Materials and Methods

### Study population

Over a span of three year, from January 2004 to December 2006, we selected a total of 1,075 consecutive subjects (803 men and 272 women) who underwent coronary arteriography for suspected or known coronary atherosclerosis at the First Affiliated Hospital of Nanjing Medical University (Nanjing, China). Patients with a history of hyperlipidemia, cerebrovascular disease, peripheral arterial disease, infectious processes within the 2 weeks preceding catheterization heart failure (Killp Class≥2 after acute myocardial infarction), or severe hepatic or renal disease were excluded. Written informed consent was obtained from all enrolled participants and this study was approved by the Ethics Committee of the First Affiliated Hospital of Nanjing Medical University.

### Biochemical analysis

Twelve-hour fasting blood samples were collected from all enrolled subjects. All measurements were conducted at the clinical laboratory in the First Affiliated Hospital of Nanjing Medical University. Total cholesterol (TCH, mmol/L), triglyceride (TG, mmol/L), fasting high-density lipoprotein-cholesterol (HDL-C; mmol/L), fasting low-density lipoprotein-cholesterol (LDL-C, mmol/L), blood urea nitrogen (BUN, mmol/L) and creatinine (CR, µmol/L) levels were determined by enzymatic procedures on an automated autoanalyzer (AU 2700 Olympus, First Chemical Ltd, Tokyo, Japan).

### Coronary angiography

Coronary angiography was carried out according to the Judkins technique, and images of the right and left coronary trees were obtained with routine standardized projections and recorded on Kodak 3-mm cinefilm (New York) at a rate of 30 frames per second. All coronary angiograms were evaluated by two experienced cardiologists who were blinded to the laboratory results of the patients. The severity of each lesion was assessed by quantitative coronary angiography.

### Scoring of coronary angiogram

Coronary angiograms were scored according to vessel score and Gensini's score. Vessel score was defined as the number of vessels with significant stenosis (≤50% of lumen diameter patency). Scores ranged from 0 to 3, depending on the number of vessels involved. In Gensini's scoring system [8], angiographic stenosis in the range of 0%–25% was scored as 1 point, stenosis in the range of 25%–50% was scored as 2 points, 50%–75% was scored as 4 points, 75%–90% was scored as 8 points, 90%–99% was scored as 16 points, and total occlusion was scored as 32 points. Each stenosed segment was then weighted from 0.5 to 5, depending on the functional significance of the area supplied by that segment. These scores were multiplied by the coefficient defined for each coronary artery and segment, and the results were then summed, According to this system, a substantial reduction in lumen diameter was assigned a higher score than a distal lesion.

### Anthropometric measurements

Anthropometric measurements were taken while the patients were barefoot and donned an examining gown. Body mass index (BMI) was calculated as body weight (kg) divided by the square of height (m^2^). Blood pressure was measured in the bare right arm with the participant seated. Three readings were obtained for each participant, and the mean was recorded for each patient.

### Cigarette smoking and alcohol intake

Cigarette smoking (number of cigarettes per day) and alcohol (amount per week) intake were assessed by means of standardized questionnaire. For smoking, patients were classified as non-smokers (never having smoked) or smokers (including former and current smoking). Patients who smoked ≥1 cigarette per day during preceding year were classified as current smokers. For alcohol use, patients were classified as non-drinkers (never having consumed alcohols) or drinkers (including former and current drinking). Patients who consumed at least 50 g per week of alcoholic beverages were regarded as current drinkers.

### Hypertension and diabetes mellitus

Patients were defined as having hypertension if they had been previously diagnosed with hypertension and took antihypertensive drugs or if their blood pressure levels were ≥140 mmHg in systole or ≥90 mmHg in diastole. Patients were defined as having diabetes mellitus if they had been previously diagnosed with diabetes mellitus and had received oral hypoglycemic agents or insulin therapy, if their fasting blood sugar (FBS) levels were above 126 mg/dl, or their postprandial 2 h blood glucose (PP2hr glucose) levels were above 200 mg/dl [9].

### Family history of coronary artery disease

All participants completed a detailed self-administered family history questionnaire. The following information was collected and analyzed for all first-degree (parents, siblings, and offspring) family members, including current age or age at death, history of heart attack, age at heart attack, and whether or not hospitalized for a heart attack [10].

### Single nucleotide polymorphism (SNP) genotyping

Genomic DNA was extracted from the patients' peripheral blood leucocytes by a common salting-out procedure using peripheral blood samples anticogulated with ethylenediaminetetraacetic acid (EDTA). SNP genotyping was performed using the 5′ nuclease discrimination assay (Taqman Assays, Applied Biosystems, and Foster City, CA, USA) on an ABI PRISM 7900HT Sequence Detection System. The quality of SNP genotyping was verified by direct DNA sequence analysis of 1075 samples.

### Statistical analysis

Data were analyzed using Statistics Package for Social Sciences software (SPSS, version 16.0; Chicago, IL, USA). Patients were classified into two groups according to their Gensini scores using the median as a cutoff point: ≥22 for the high score group and <22 for the low score group. Genotypes were tested for Hardy-Weinberg equilibrium among all participants using a χ^2^ test with one degree of freedom. Variables were tested for normality with Kolmogorov-Smirnov statistics. Data of BMI were normally distributed and therefore presented in the form of mean ± SD, whereas skewed data, including AGE, SBP, DBP, TCH, TG, HDL-C, LDL-C, BUN, CR and Gensini's score were expressed as medians and interquartile ranges. Differences in selected demographic continuous variables among groups were evaluated by using one-way analysis of variance (ANOVA) or the Kruskal-Wallis H test. Categorical variables, namely sex, smoking status, alcohol consumption status, hypertension status, diabetes status and family history of CHD were compared among the genotypes of rs6903956 using a χ2-test. Variables of counts of diseased vessel were compared among the genotypes of rs6903956 using a χ2-test for linear-by-linear association. A binary logistic regression procedure was used for association analyses of Gensini score-related phenotypes, association of rs6903956 genotype variants and angiographical characteristics of coronary atherosclerosis risk were estimated by computing the *OR* and their 95% *CI* without/with adjustments for age, sex, BMI, and other confounding risk factors. The genotype data were further stratified by age, sex, BMI, and smoking subgroups. Differences were considered to be significant if the null hypothesis could be rejected with >95% confidence. All *p* values are two-tailed.

### Power calculation

Power calculation was performed using the NCSS/PASS Dawson edition software (Kaysvile, UT) for Windows XP. A logistic regression of a binary response variable (Y) on a binary independent variable (X) with a sample size of 1075 observations (of which 50% are in the group X = 0 and 50% are in the group X = 1) achieves 78% power at a 0.05 significance level to detect a change in Prob (Y = 1) from the baseline value of 0.138 to 0.200. This change corresponds to an OR of 1.564.

## Results

### Genetic characteristics of the study cohort

A total of 1075 ethnic Han Chinese participants were genotyped for the rs6903956 SNP and included in this study. The clinical characteristics of the population according to genotype are summarized in Table 1. The rs6903956 genotype groups did not differ significantly in their demographics, biochemical laboratory test result, family history, or lifestyle habits. However, Gensini score did differ significantly among the rs6903956 genotypes (*p* = 0.025).

**Table 1:** Patient characteristics by rs6903956 genotype.

Variables	Rs6903956 genotype			Statistical parameter	*P*-value
	GG	AG	AA		
**AGE** (years)	63.00(54.00∼70.00)	62.00(53.00∼69.25)	64.00(60.00∼69.00)	0.231	0.891
**SBP** (mmHg)	130(120∼145)	130(120∼140)	130(120∼140)	0.274	0.872
**DBP** (mmHg)	80(70.00∼85.00)	80(70.00∼88.50)	80(70.00∼90.00)	0.567	0.753
**BMI**(kg/m^2^)	24.98±3.07	24.85±3.11	24.06±3.01	0.626	0.535
**CH**(mmol/L)	4.08(3.47∼4.59)	4.13(3.63∼4.70)	3.68(3.44∼4.81)	2.162	0.339
**TG**(mmol/L)	1.42(1.00∼2.02)	1.31(0.94∼2.00)	1.19(0.84∼1.45)	7.890	0.019
**HDL-C**(mmol/L)	0.98(0.85∼1.16)	1.02(0.86∼1.18)	0.99(0.78∼1.27)	0.869	0.648
**LDL-C**(mmol/L)	2.37(1.89∼2.85)	2.44(2.02∼3.05)	2.37(2.06∼3.00)	4.300	0.116
**BUN**(mmol/L)	5.32(4.35∼6.51)	5.31(4.37∼6.24)	4.51(4.15∼4.91)	4.449	0.108
**CR**(mmol/L)	73(61∼87)	75(62∼92)	64(55∼78)	1.716	0.424
**Gensini score**	20.00(2.00∼57.00)	28.50(3.75∼71.25)	32.00(23.00∼68.00)	7.403	0.025
**Sex(male/female)**	663/230	130/39	10/3	0.574	0.750
**Smoking status (Yes/No)**	413/480	91/78	5/8	3.707	0.157
**Alcohol consumption (Yes/No)**	192/701	36/133	3/10	0.023	0.989
**Hypertension status (Yes/No)**	584/308	112/57	8/5	0.338	0.987
**Diabetes status (Yes/No)**	155/738	23/146	0/13	4.056	0.132
**Family history of CHD(Yes/No)**	103/790	27/14	2/11	2.721	0.257

Data shown in the table: systolic blood pressure (SBP, mmHg), diastolic blood pressure (DBP, mmHg), body mass index (BMI, kg/m^2^), total cholesterol (CH, mmol/L), triglyceride (TG, mmol/L), fasting high-density lipoprotein-cholesterol (HDL-C; mmol/L), fasting low-density lipoprotein-cholesterol (LDL-C, mmol/L), blood urea nitrogen (BUN, mmol/L), creatinine (CR, µmol/L) and CHD (coronary heart disease).

The observed genotypic frequencies were consistent with Hardy-Weinberg equilibrium (Table 2, *p* = 0.124) with a risk allele frequency of 0.09 (observed frequency of allele A).

**Table 2:** Hardy-Weinberg equilibrium test for rs6903956 genotype frequencies among all patients (N = 1075).

comparator		Rs6903956 genotype		Allele	
	GG n (%)	AG n (%)	AA n (%)	G n (%)	A n (%)
**Observed frequencies**	893(83.07)	169(15.72)	13(1.21)	1955(90.93)	195(9.07)
**Expected frequencies**	888.84	177.32	8.84	—	—

χ2 = Σ (observed frequencies-expected frequencies) ^2^/expected frequencies = 2.363, df = 1, *P* = 0.124, rs6903956 genotype information was available for 1075 patients.

### Genotype distribution of rs6903956 polymorphisms in subjects grouped by diseased vessel count

The distribution of rs6903956 polymorphism genotypes within the diseased vessel score subgroups are presented by Table 3. Coronary angiography revealed coronary heart disease in 716/1075 patients, including 264 patients who had a single vessel diseases affected, 192 who had 2 vessel diseases affected and 260 who had 3 vessel diseases affected. As reported in Table 3, we observed a dose-dependent influence of the genotype of the human rs6903956 polymorphisms on the response to the vessel score (*χ2* test for linear-by-linear association; *P* = 0.024 for GG vs. AG vs. AA and *P* = 0.042 for GG vs. AG+AA, respectively).

**Table 3:** Genotype distribution of the human rs6903956 polymorphisms in subjects grouped by the vessel score.

Genotype	Diseased vessel score				linear-by-linear *χ2* value	*P*-value
	0	1	2	3		
Rs6903956, n (%)					5.074	0.024
GG	307(85.5)	222(84.1)	157(81.8)	207(79.6)		
AG	50(13.9)	38(14.4)	34(17.7)	47(18.1)		
AA	2(0.6)	4(1.5)	1(0.5)	6(2.3)		
Rs6903695, n (%)					4.116	0.042
GG	307(85.5)	222(84.1)	157(81.8)	207(79.6)		
AG+GG	52(14.5)	42(15.9)	35(18.2)	53(20.4)		

### Rs6903956 polymorphism, genotype distribution, and association of genotypes with angiographical characteristics of coronary atherosclerosis risk, in patients grouped by Gensini score

A univariate logistic regression analysis (Table 4) indicated that the frequencies of GG, AG and AA genotypes of rs6903956 in the lower (<22) Gensini score group (86.2, 13.4 and 0.4%, respectively) differed from those in the higher (≥22) Gensini score group (80.0, 18.0 and 2.0%, respectively). When the GG genotype was used as the reference group, both the heterozygote AG genotype (*p* = 0.030, *OR* = 1.444, 95% *CI* = 1.036∼2.013) and the homozygote AA (*p* = 0.021, *OR* = 5.896, 95% *CI* = 1.299∼26.750) were associated with a significantly increased risk of higher Gensini score, with the effect being more robust for the AA genotype. When we combined the heterozygote AG and homozygote AA as a pooled risk genotypes comparison group, assuming a dominant effect of the variant A allele, the combined genotype XA (AG+AA) was associated with a highly significant 56.4% increased risk of higher Gensini score (*p* = 0.007, *OR* = 1.564, 95% *CI* = 1.132∼2.162) relative to the GG genotype.

**Table 4:** Association of the rs6903956 genotypes with the angiographical characteristics of coronary atherosclerosis using univariate logistic analyse.

Genotype	Gensini score		*OR* (95%*CI*)	*P*-value
	Group1 (n = 536)	Group2 (n = 539)		
Rs6903956, n (%)				
GG	462(86.2)	431(80.0)	1	
AG	72(13.4)	97(18.0)	1.444(1.036∼2.013)	0.030
AA	2(0.4)	11(2.0)	5.896(1.299∼26.750)	0.021
Rs6903695, n (%)				
GG	462(86.2)	431(80.0)	1	
AG+GG	74(13.8)	108(20.0)	1.564(1.132∼2.162)	0.007

*OR*: odds ratio.

*CI*: confidence internal.

Group 1: the lower (<22) Gensini score group.

Group 2: the higher (≥22) Gensini score group.

Additionally, when we performed a multivariate logistic regression analysis of the relationship between rs6903956 genotype and angiographical characteristics of coronary atherosclerosis risk with age, gender, smoking status, drinking status, BMI, and family history of coronary heart disease as covariates (as shown in Table 5), We observed that the genotype distribution of the rs6903956 polymorphisms was associated significantly with the angiographical characteristics of coronary atherosclerosis risk (*p* = 0.004, *OR* = 1.578, 95% *CI* = 1.155∼2.154 for GG vs. GA vs. AA; *p* = 0.013, *OR* = 1.541, 95% *CI* = 1.097∼2.163 for GG vs. GA+AA). The covariates of age, gender, and smoking status were also associated with a higher Gensini score.

**Table 5:** Association of the risk factors with the angiographical characteristics of coronary atherosclerosis using multivariate Logistic regression analyse.

Variables	Gensini score		*OR(95%CI)*	*P*-value
	Lower(N = 536)	Higher(N = 539)		
**Rs6903956**				
**GG/AG/AA**	(462/72/2)	(431/97/11)	1.578(1.155∼2.154)	0.004
**Sex(Male/Female**	(364/172)	(439/100)	0.657(0.472∼0.914)	0.013
**Age**	61(52∼68)	66(57∼71)	1.046(1.033∼1.059)	0.000
**Smoking status(Yes/no)**	(202/334)	(307/232)	2.138(1.602∼2.855)	0.000
**Rs6903956**				
**GG/(AG+AA)**	(462/74)	(431/108)	1.541(1.097∼2.163)	0.013
**Sex(Male/Female)**	(364/172)	(439/100)	0.656(0.472∼0.914)	0.013
**Age**	61(52∼68)	66(57∼71)	1.046(1.033∼1.059)	0.000
**Smoking status(Yes/no)**	(202/334)	(307/232)	2.134(1.599∼2.848)	0.000

*OR*: Odds ratio.

*CI*: confidence internal.

### Association analyses in stratified subgroups

We further analysed the associations of the rs6903956 polymorphism with the risk of higher Gensini score with the patients stratified by age, sex, BMI, smoking status, and drinking atatus. As indicated in Table 6, the frequency distribution of the rs6903956 mutant was differed in the higher Gensini score subjects than it in the lower Gensini score subjects belonging to high-risk category as male (*p* = 0.023, *OR* = 1.579, 95% *CI* = 1.064∼2.344 for rs6903956 AG; *p* = 0.005, *OR* = 1.753, 95% *CI* = 1.189∼2.586 for combined rs6903956 (AG+AA)), smokers (*p* = 0.005, *OR* = 2.075, 95% *CI* = 1.249∼3.448 for rs6903956 AG; *p* = 0.002, *OR* = 2.233, 95% *CI* = 1.350∼3.694 for combined rs6903956 (AG+AA)), However, the risk factors of being older than 60 years of age, having a BMI≥24 kg/m^2^, and being a drinker were not associated with a greater likelihood of having a higher Gensini score.

**Table 6:** The risk assessment of rs6903956 genotype to the coronary atherosclerosis.

Variables	Rs6903956 genotype			Adjusted *OR(95%CI)*	*P*-value
	Genotype	GS = 0–21.9 Group 1(%)	GS> = 22 Group 2(%)		
**Age (years)**					
**<60**	GG	211(50.7)	135(32.5)	1	
	AG	33(7.9)	34(8.2)	0.268(0.022∼3.223)	0.300
	AA	1(0.2)	2(0.5)	0.423(0.034∼5.278)	0.504
	AG+AA	34(8.2)	36(8.7)	1.633(0.962∼2.772)	0.069
**> = 60**	GG	251(38.1)	296(44.9)	1	
	AG	39(5.9)	63(9.6)	1.385(0.887∼2.162)	0.152
	AA	1(0.2)	9(1.4)	7.680(0.946∼62.377)	0.056
	AG+AA	40(6.1)	72(10.9)	1.541(0.999∼2.376)	0.051
**BMI(kg/m^2^)**					
**<24**	GG	157(39.4)	170(42.7)	1	
	AG	25(6.3)	39(9.8)	1.584(0.890∼2.891)	0.118
	AA	1(0.3)	6(1.5)	5.3412(0.628∼45.471)	0.125
	AG+AA	26(6.5)	45(11.3)	1.749(1.003∼3.051)	0.049
**> = 24**	GG	301(45.2)	255(38.3)	1	
	AG	47(7.1)	58(8.7)	1.424(0.927∼2.188)	0.107
	AA	1(0.2)	4(0.6)	5.347(0.555∼51.487)	0.147
	AG+AA	48(7.2)	62(9.3)	1.498(0.982∼2.284)	0.061
**Sex(male/female)**					
**Male**	GG	315(39.2)	348(43.3)	1	
	AG	49(6.1)	81(10.1)	1.579(1.064∼2.344)	0.023
	AA	0(0)	10(1.2)	—	—
	AG+AA	49(6.1)	91(11.3)	1.753(1.189∼2.586)	0.005
**Female**	GG	147(50.4)	83(30.5)	1	
	AG	23(8.5)	16(5.9)	1.122(0.550∼2.291)	0.752
	AA	2(0.7)	1(0.4)	0.814(0.068∼9.684)	0.870
	AG+AA	25(9.2)	17(6.2)	1.098(0.549∼2.196)	0.792
**Smoking status**					
**No**	GG	285(50.4)	195(34.5)	1	
	AG	47(8.3)	31(5.5)	0.975(0.585∼1.625)	0.922
	AA	2(0.4)	6(1.1)	3.688(0.669∼20.335)	0.134
	AG+AA	49(8.7)	37(6.5)	1.087(0.667∼1.772)	0.738
**Yes**	GG	177(34.8)	236(46.4)	1	
	AG	25(4.9)	66(13)	2.075(1.249∼3.448)	0.005
	AA	0(0)	5(1)	—	—
	AG+AA	25(4.9)	71(13.9)	2.233(1.350∼3.694)	0.002
**Drinking status**					
**No**	GG	366(43.4)	335(39.7)	1	
	AG	59(7.0)	74(8.8)	1.424(0.965∼2.103)	0.075
	AA	2(0.2)	8(0.9)	3.790(0.743∼19.349)	0.019
	AG+AA	61(7.2)	82(9.7)	1.504(1.028∼2.199)	0.036
**Yes**	GG	96(41.6)	96(41.6)	1	
	AG	13(5.6)	23(10.0)	1.755(0.836∼3.685)	0.137
	AA	0(0)	3(1.3)	—	—
	AG+AA	13(5.6)	26(11.3)	1.990(0.961∼4.120)	0.064

Adjusted for age, sex, BMI in a binary logistic regression for each stratum, data are shown as numbers (percentages are given in parentheses).

GS: Gensini score, Group 1(N = 536), Group 2 (N = 539).

## Discussion

It is well established that CAD, a complex multifactorial disease, is one the most common causes of death in China. Atherosclerosis is the most common cause of mortality and morbidity of CAD [11]. We all know that CAD is associated with hypertension, obesity, low HDL-C levels and diabetes mellitus (DM) [12]–[16]. Hence, identification the pathogenesis of genetic factor for CAD is of extremely importance.

The present study evaluated the association between angiographical characteristics of coronary atherosclerosis and the rs6903956 polymorphism in an ethnic Han Chinese population. In a cohort of 1,075 consective patients (803 men and 272 women) who underwent coronary arteriography for suspected or known coronary atherosclerosis, we found that severity of the coronary atherosclerosis estimated by Gensini score and diseased vessel counts both differed in relation to rs6903956 genotype. However, the traditional risk factors of CAD (ie age, blood pressure, total cholesterol, triglyceride levels, etc.) did not differ among the three rs6903956 genotypes.

In the first GWAS for CAD in an ethnic Han Chinese population, Dandona et al. identified rs6903956 on chromosome 6p24.1 as being significantly associated with susceptibility to CAD [6], although the exact mechanism underlying the relationship between rs6903956 and CAD is still unclear, coronary arteriography remains the golden standard for CAD.

Our present study was conducted to evaluate the association between the angiographical characteristics of coronary atherosclerosis and the rs6903956 polymorphism in the Chinese population. In our study, the Gensini score system was used to define the angiographical characteristics of coronary atherosclerosis. We found that the prevalence rates of the heterozygous AG genotype and the homozygous AA genotype were significantly higher among higher Gensini score subjects than among lower Gensini score subjects, and this difference reached statistical significance. This is the first study, however, to explore the association between rs6903956 genotype and severity of the coronary atherosclerosis as defined by Gensini scores system.

Considering that one-third of all cigarettes are smoked in China [17], advanced age, alcohol consumption, high BMI, and male gender are all traditional risk factors to CAD, so our study included a deeper analysis of the relationship between rs6903956 genotype and the risk of higher Gensini score stratified by age, sex, BMI, smoking, and drinking status .We found that being a smoker or being male was associated with an increased likelihood of being in the higher Gensini score group. This relationship with smoking fits well with previous research showing that the number of cigarettes smoked was an independent risk factor for mortality in subjects with atherosclerosis in Chinese population [18]. Maleness has previously been associated with risk of coronary artery disease in subjects of European ancestry [19]. Interestingly, several traditional risk factors for CAD, such as being older than 60 years of age, being a drinker, and having a BMI≥24 kg/m^2^, were not found to be signifincalty associated with a higher Gensini score in our study. The lack of positive associations for these factors could have been due to the effects being too weak to be confirmed in the present sample size or our enrolled patients not adequately representing the general population.

In addition to the rs6903956 SNP examined here, another SNP, rs499818, which is located in the same chromosomal region 6p24.1, has been demonstrated to be associated with coronary heart disease. A community-based genome-wide association study of major cardiovascular disease outcomes was conducted, 1345 Framingham Heart Study participants from the largest 310 pedigrees were recruited in the study, and, 70,987 qualifying SNPs (Affymetrix 100 K GeneChip) were analyzed. The results demonstrated that the rs499818 was associated with the major cardiovascular outcomes (*p* = 6.6×10(−6)) [20]. In the PAGE study, another GWAS identified several SNPs associated with incident coronary heart disease, the rs499818 SNP to be associated with the incidence of CAD in white American participants. (P = 0.0002, Cox proportional hazards model with additive mode of inheritance adjusted for age, sex, and ancestry as needed) [21], although rs499818 and rs6903956 are located in the same chromosomal region 6p24.1, the distance between them was observed to be greater than 1 M. Future studies are need to determine the linkage disequilibrium between these two SNPs and to further examine whether these SNPs are indeed a causal variant or just a marker/tagger.

## Conclusion

In conclusion, the present findings demonstrating that the A allele of the rs6903956 SNP confers greater risk of CAD, and a G-to-A allele substitution may underlie the relationship between rs6903956 and CAD. Although the exact biological mechanism of this association remains to be explored, our study provides credible evidence that the rs6903956 polymorphism may contribute to the etiology of the severity of coronary atherosclerosis and plays an important role in the atherosclerotic process in the Chinese population. Further studies are needed to interpret SNP rs6903956 on coronary atherosclerosis susceptibility.
